# Editorial: Emotion regulation and mental health in older adults

**DOI:** 10.3389/fpsyg.2023.1173314

**Published:** 2023-03-24

**Authors:** Gary Christopher, David Facal

**Affiliations:** ^1^Swansea University, Swansea, United Kingdom; ^2^Department of Developmental and Educational Psychology, University of Santiago de Compostela, Santiago de Compostela, Spain

**Keywords:** emotion regulation, mental health, older adult, gerontology, cultures and ethnicities

Emotion regulation describes a process enabling us to respond appropriately to the vicissitudes of life. Without the ability to control and harness emotions effectively, we are at the mercy of trials and tribulations of our daily lives. Because of this, poor emotion regulation is linked to several health and mental health conditions. This interplay between physical and mental health is crucial when considering the quality of life of older adults. Of interest, given that aging is generally associated with decline and loss—an inaccurate and misleading stereotype that feeds ageist assumptions—older adults tend to be more adept at regulating their emotions. This can be seen as a way to compensate for changes (Urry and Gross, 2010). This manifests in several ways, including a smaller yet more intimate social circle (Carstensen et al., 2003) and active mood repair through a bias toward positive information (Isaacowitz et al., 2008).

With a growing expectation to lead a long and healthy life, emotional resilience is of paramount importance. The papers in this issue offer a snapshot of how research into emotion regulation in later life encompasses all aspects of our lives. Pfluger et al. from Switzerland demonstrate that emotion regulation strategies mediate between exposure to complex trauma early in life and the development of internalizing mental health disorders across the lifespan, such that less adaptive strategies—in this case, emotion suppression—are associated with depression and anxiety later in life.

Sleep quality significantly influences how effectively we function in our daily lives and, in particular, how poor sleep quality negatively impacts wellbeing. We know depression and anxiety are intimately linked to perceived quality of life. We also know that sleep quality significantly impacts physical and mental health. The study by Kennair et al. shed light on the complex interrelationship between these factors using a sample of older adults from Norway. Sleep is again the focus of the paper by Zhang et al. from China. They explored the link between sleep quality and subjective wellbeing in older adults, showing a significant effect mediated by negative emotions and indirectly moderated by perceived social support.

Our perceived place in society and our roles remain significant in later life. A combination of loneliness and an absence of mutually caring relationships, a situation referred to as thwarted belongingness, is explored by Yu in the context of successful aging among older adults in China. Meaning in life acts to buffer the negative relationship *via* positive mental health. The security and solidity of relationships become increasingly significant as we age. Kieslich and Steins explore how German older adult couples who have been together for many years manage stress. They identify the need to foster emotional attachment between partners to mitigate conflict when it arises. Internet use is constantly rising in this age group. This activity is often seen in a negative light, especially when it comes to mental health. The paper by Li and Yang adds to the complexity by exploring the role social capital plays in all this within the Chinese population.

Changes in our health require us to reconsider our self-image. Issues with continence in later life is often physically and mentally challenging, impacting negatively on quality of life in many instances. In a study from Iran, Javanmardifard et al. explore how the taboo associated with this condition can be confronted, and so encourage people to get support.

In the case of people living with dementia, such knowledge that this diagnosis brings requires much reassessment. Over time, activities which, throughout their lives, promised pleasure require increasing effort. Many things influence a person's engagement in leisure activities. A decline in physical and mental capabilities is a significant determiner for people living with dementia. Park and Kim, in a study from South Korea, identify gender as a significant predictor of the type of activity preferred. They discuss their findings in relation to activity programmes in care home settings.

Cultural differences in self-regulatory behavior mean that it is essential for measures to be appropriately validated to ensure they retain the essence of the original yet are sensitive to different beliefs and expectations. It is also crucial that such tools are relevant to specific age groups. Motamed-Jahromi et al. adapt a measure of self-regulation for Persian-speaking older adults.

Although change is inevitable, we must reconsider our assumptions about aging. The concept of healthy or successful aging is often unhelpful as such terms assume some arbitrary benchmark which, if not reached, implies failure. Instead, we need to acknowledge that later life can be associated with much that is positive, rewarding, and fulfilling. It is not about avoiding change, rather, we should embrace it and muster our psychological resources to meet the challenge head-on and build resilience. Nostalgia offers one way to boost vital psychological resources. It can help people maintain psychological wellbeing in the face of threat and offers an effective regulatory mechanism. Fleury et al. present a mini review of the literature on how feeling safe is essential to our understanding of aging, and the potential role nostalgia can play in facilitating this.

This issue brings together a range of research from different countries, offering valuable insights into the role of emotion regulation from different cultural viewpoints. This is vital as we need to understand aging from the unique perspectives of the societies in which people live. Views on aging differ. How people express emotion differs. The types and relevance of support differ. We need to move away from universal to more dynamic and adaptive assumptions about aging.

## Author contributions

All authors listed have made a substantial, direct, and intellectual contribution to the work and approved it for publication.
